# The role of radiotherapy in pulmonary large cell neuroendocrine carcinoma: propensity score matching analysis

**DOI:** 10.1093/jrr/rraa036

**Published:** 2020-06-22

**Authors:** Ling Cao, Hong-Fen Wu, Ling Zhao, Yan Bai, Zhi-lan Jiang, Wan-Ju Yang, Shi-xin Liu

**Affiliations:** Department of Radiation Oncology, Jilin Provincial Cancer Hospital, Changchun, 130012, China

**Keywords:** pulmonary large cell neuroendocrine carcinoma, radiotherapy, survival, surveillance, epidemiology, and end results (SEER)

## Abstract

The aim of the study was to investigate the survival advantage of radiotherapy (RT) in patients with pulmonary large cell neuroendocrine carcinoma (LCNEC). Patients with pulmonary LCNEC were extracted from the Surveillance, Epidemiology, and End Results (SEER) dataset between January 2004 and December 2013. Propensity score matching (PSM) analysis with 1:1 was used to ensure well-balanced characteristics of all comparison groups. A total of 1480 eligible cases were identified, with a median follow-up time of 11 months (0–131 months). After PSM, 980 patients were classified in no radiotherapy (No RT) and radiotherapy (RT) groups (*n* = 490 each). Patients in the RT group harbored significantly higher 3- and 5-year overall survival (OS) and cancer-specific survival (CSS) rates compared to those in the No RT group (both *P <* 0.05). Furthermore, RT was an independent favorable prognostic factor of OS as well as CSS in multivariate analysis, both before [OS: hazard ratio (HR) 0.840, 95% confidence interval (CI) 0.739–0.954, *P* = 0.007; CSS: HR 0.847, 95% CI 0.741–0.967, *P* = 0.014] and after (OS: HR 0.854, 95% CI 0.736–0.970, *P* = 0.016; CSS: HR 0.848, 95% CI 0.735–0.978, *P* = 0.023) PSM. In subgroup analysis, American Joint Committee on Cancer (AJCC) stage II and III, tumor size 5-10 cm, patients who underwent no surgery, or patients who received chemotherapy could significantly benefit from RT (all *P* < 0.05). To sum up, our findings suggested that RT could prolong the survival of patients with pulmonary LCNEC, especially those with stage II and III, tumor size 5-10 cm, those with no surgery, or those who received chemotherapy.

## INTRODUCTION

Pulmonary large cell neuroendocrine carcinoma (LCNEC) is an uncommon malignancy, accounting for ~3% of all lung cancers [1]. In 1991, Travis *et al*. suggested that the large cell neuroendocrine tumor was a new type of solitary pulmonary neuroendocrine tumor, which was different from typical, atypical carcinoid and small cell carcinoma [2]. Then, in 1999 and 2004, the World Health Organization (WHO) concluded that pulmonary LCNEC was a variant of large cell carcinoma, which was among the pulmonary neuroendocrine tumors, and belonged to non-small cell lung cancer (NSCLC) [3, 4].

Due to its low incidence and inadequate relevant clinical trial data, there is a lack of clinical understanding of the biological characteristics of pulmonary LCNEC [1, 5]. Furthermore, the role of radiotherapy (RT) in the treatment of local or advanced pulmonary LCNEC is still unclear, but some authors suggest its use in locally advanced disease setting [6, 7]. However, as far as we know, there is no large-population-based report on the role of RT in pulmonary LCNEC, and small-scale studies have yielded conflicting results [1]. Based on this, the present study aimed to investigate the survival advantage of RT in patients with pulmonary LCNEC.

## MATERIALS AND METHODS

### Ethics statement

The National Cancer Institute’s Surveillance, Epidemiology, and End Results (SEER) program, initiated from 1973 and annually updated, utilizes population-based data to develop comprehensive sources [8], covering ~30% of the US population [9]. The SEER Research Data Agreement was signed for access to SEER information under reference number 16462-Nov2016. The data were obtained following strictly approved guidelines. The data, which had been obtained by the United States Department of Health and Human Services, was considered by the Office for Human Research Protection to be on non-human subjects as they were publicly available and de-identified. Thus, the study did not require approval by the institutional review board.

### Study population

The SEER*State v8.3.5 tool, released on 6 March 2018, was used to determine and select eligible patients. The study duration ranged from January 2004 to December 2013. The inclusion criteria were as follows: age at diagnosis ≥20 years; LCNEC pathologically confirmed based on histology (ICD-O-38013/3); restriction on site recodes ICD-O-3/WHO 2008 (International Classification of Diseases for Oncology, Third Edition) to ‘Lung and Bronchus’. The exclusion criteria were as follows: (i) age at diagnosis ≤ 20 years; (ii) patients with >1 primary malignancy; (iii) patients with low-grade pathology (Grade I and Grade II) because LCNEC is a type of high-grade neuroendocrine lung tumor; (iv) patients without pathological diagnosis based on histology; (v) patients without prognostic data; (vi) patients without complete important clinicopathological data, including marital status, race, primary tumor site, 6th American Joint Committee on Cancer (AJCC) tumor stage and specific surgical type; (vii) patients who died within 3 months after surgery. The remainder were defined as the SEER primary cohort.

### Covariates

The following demographic and clinicopathological data were collected from the SEER dataset: age at diagnosis, marital status, race, gender, primary location, laterality, grade, tumor size, AJCC stage, surgery, chemotherapy, RT and follow-up information. Continuous variables (age and tumor size) were converted into categorical ones based on well-defined cut-off values. We used the AJCC staging system 6th edition, and we limited our research to between 2004 and 2013, because this edition was published in 2004.

The primary endpoint was overall survival (OS), while cancer-specific survival (CSS) was the secondary endpoint. OS refers to the duration from diagnosis to the most recent follow-up date or date of death. CSS refers to the time duration from diagnosis to the most recent follow-up date or date of death caused by pulmonary LCNEC. There was a predetermined cut-off date based on the SEER 2016 submission database, containing death information until 2014. The cut-off date was 31 December 2014 in our study.

### Propensity score matching

Selection bias might be unavoidable in observational research, leading to unevenly distributed confounding factors between two groups. Propensity score matching (PSM) is the conditional probability of assignment to a particular treatment given a vector of observed covariates [10]. Therefore, PSM was utilized in the present research to reduce selection bias and imbalanced distributions of the confounding factors [11]. In this study, PSM was performed between the RT group and the No RT group of each subgroup. Propensity scores for all patients were estimated using a logistic regression model, which included all covariates that might have affected patients survival except RT (age at diagnosis, sex, race, marital status, primary site, laterality, grade, tumor size, AJCC stage, surgery and chemotherapy). The PSM plug-in of SPSS software was utilized to determine the propensity score in each case. Afterwards, PSM was performed using 1:1 nearest neighbor matching with a caliper of 0.01 for the acceptance of a matched pair.

### Statistical analyses

Continuous data are shown as mean with standard deviation (SD) and categorical variables are presented as numbers with percentages. Pearson’s χ2 or Fisher’s exact tests were utilized to compare the clinicopathological characteristics before and after PSM. The Kaplan–Meier method was employed to determine patients’ survival rates, along with log-rank tests for assessing differences among groups. Both univariate and multivariate Cox proportional hazards regression analyses were used to examine the correlations between diverse factors and survival. To be specific, variables of potential significance in univariate analysis (*P* < 0.05) or previously reported to be prognostic factors were enrolled in multivariate analysis. SPSS software (SPSS Inc., Chicago, USA, version 23) was used for statistical analysis and *P* < 0.05 was considered as statistically significant.

## RESULTS

### Patient characteristics before and after PSM

**Fig. 1.**
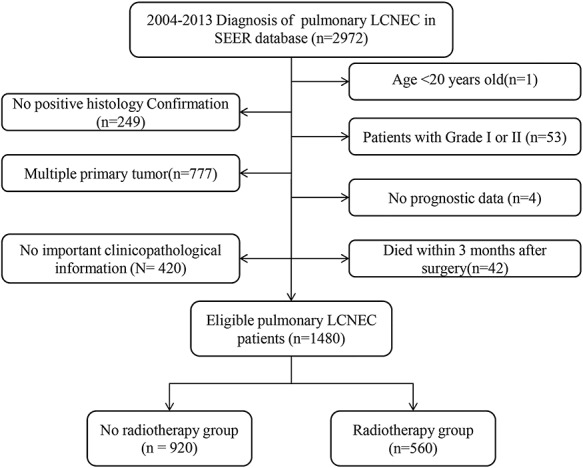
Flow chart for screening eligible patients.

In total, 1480 cases were enrolled in this research. Patients were further divided into a No RT (*n* = 920) and an RT (*n* = 560) group, and the specific screening process is shown in Fig. 1.The distribution of patient characteristics in both groups are displayed in Table 1. The median follow-up time was 11 months (0–131 months). In terms of survival rates, the 1-, 3- and 5-year OS and CSS were 47.7, 23.5 and 17.3% and 50.5, 27.0 and 21.2%, respectively. The following variables: marital status, race, sex, age, primary site, grade, laterality, tumor size, AJCC stage, surgery and chemotherapy were included in PSM. After PSM at a 1:1 ratio, both No RT and RT groups comprised 490 patients, respectively. Despite the significant differences in certain variables (age, primary site, chemotherapy), no significant differences were observed in grade (*P* = 0.385), tumor size (*P* = 0.162), AJCC stage (*P* = 0.635) and surgery (*P* = 0.420) after PSM (Table 1).

**Table 1:** Baseline characteristics before and after propensity score matching

Variable	Before PSM			After PSM		
	No RT (*n* = 920)	RT (*n* = 560)	*P*	No RT (*n* = 490)	RT (*n* = 490)	*P*
Age, years			0.008			<0.001
<60	236 (25.65%)	182 (32.50%)		116 (23.67%)	162 (33.06%)	
≥60 and <80	589 (64.02%)	336 (60.00%)		315 (64.29%)	293 (59.80%)	
≥80	95 (10.33%)	42 (7.50%)		59 (12.04%)	35 (7.14%)	
Sex			0.733			0.401
Male	516 (56.09%)	309 (55.18%)		287 (58.57%)	274 (55.92%)	
Female	404 (43.91%)	251 (44.82%)		203 (41.43%)	216 (44.08%)	
Race			0.795			0.890
White	773 (84.02%)	467 (83.39%)		408 (83.27%)	410 (83.67%)	
Black	114 (12.39%)	69 (12.32%)		62 (12.65%)	58 (11.84%)	
Other	33 (3.59%)	24 (4.29%)		20 (4.08%)	22 (4.49%)	
Marital status			0.306			0.063
Married	494 (53.70%)	316 (56.43%)		249 (50.82%)	278 (56.73%)	
Unmarried	426 (46.30%)	244 (43.57%)		241 (49.18%)	212 (43.27%)	
Primary site			0.102			0.015
Main bronchus	36 (3.91%)	26 (4.64%)		24 (4.90%)	26 (5.31%)	
Upper lobe	550 (59.78%)	368 (65.71%)		276 (56.33%)	325 (66.33%)	
Middle lobe	56 (6.09%)	26 (4.64%)		31 (6.33%)	22 (4.49%)	
Lower lobe	262 (28.48%)	129 (23.04%)		147 (30.00%)	106 (21.63%)	
Overlapping lesion	16 (1.74%)	11 (1.96%)		12 (2.45%)	11 (2.24%)	
Grade			<0.001			0.385
Poorly differentiated	389 (42.28%)	177 (31.61%)		161 (32.86%)	151 (30.82%)	
Undifferentiated	126 (13.70%)	58 (10.36%)		63 (12.86%)	53 (10.82%)	
Unknown	405 (44.02%)	325 (58.04%)		266 (54.29%)	286 (58.37%)	
Laterality			0.655			0.174
Bilateral	2 (0.22%)	0 (0.00%)		2 (0.41%)	0 (0.00%)	
Left	373 (40.54%)	233 (41.61%)		216 (44.08%)	198 (40.41%)	
Right	545 (59.24%)	327 (58.39%)		272 (55.51%)	292 (59.59%)	
Tumor size			<0.001			0.162
<2 cm	181 (19.67%)	66 (11.79%)		59 (12.04%)	53 (10.82%)	
≥2 cm and <5 cm	436 (47.39%)	231 (41.25%)		219 (44.69%)	203 (41.43%)	
≥5 cm and <10 cm	173 (18.80%)	161 (28.75%)		118 (24.08%)	141 (28.78%)	
≥10 cm	34 (3.70%)	42 (7.50%)		29 (5.92%)	40 (8.16%)	
Unknown	96 (10.43%)	60 (10.71%)		65 (13.27%)	53 (10.82%)	
AJCC stage			<0.001			0.635
IA	184 (20.00%)	17 (3.04%)		19 (3.88%)	17 (3.47%)	
IB	162 (17.61%)	29 (5.18%)		26 (5.31%)	29 (5.92%)	
IIA	23 (2.50%)	5 (0.89%)		6 (1.22%)	5 (1.02%)	
IIB	51 (5.54%)	22 (3.93%)		29 (5.92%)	21 (4.29%)	
IIIA	56 (6.09%)	85 (15.18%)		44 (8.98%)	58 (11.84%)	
IIIB	84 (9.13%)	81 (14.46%)		75 (15.31%)	65 (13.27%)	
IV	360 (39.13%)	321 (57.32%)		291 (59.39%)	295 (60.20%)	
Surgery			<0.001			0.420
No surgery	446 (48.48%)	455 (81.25%)		372 (75.92%)	387 (78.98%)	
Segmentectomy/wedge resection	91 (9.89%)	29 (5.18%)		39 (7.96%)	28 (5.71%)	
Lobectomy/bilobectomy	342 (37.17%)	61 (10.89%)		67 (13.67%)	60 (12.24%)	
Pneumonectomy	41 (4.46%)	15 (2.68%)		12 (2.45%)	15 (3.06%)	
Chemotherapy			<0.001			<0.001
No/unknown	544 (59.13%)	151 (26.96%)		232 (47.35%)	145 (29.59%)	
Yes	376 (40.87%)	409 (73.04%)		258 (52.65%)	345 (70.41%)	

### CSS and OS after PSM

**Fig. 2.**
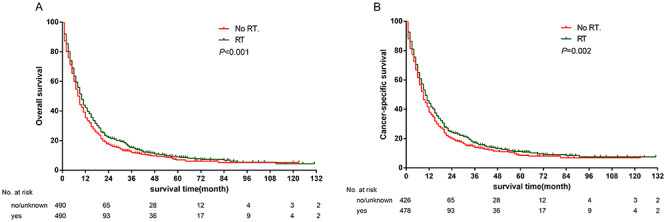
Comparison of the survival rates between No RT and RT groups after PSM. (**A**) OS; (**B**) CSS.

After PSM, the 1-, 3- and 5- year OS rates were higher in the RT group compared to the No RT groups (41.2, 14.8 and 8.8% vs 30.9, 11.2 and 6.1%, respectively; *P <* 0.001) (Fig. 2A). Consistently, the 1-, 3- and 5-year CSS rates were significantly higher in the RT group than in the No RT group (42.9, 16.6 and 11.0% vs 33.9, 12.1 and 7.6%, respectively; *P =* 0.002) (Fig. 2B).

### Prognostic factors for survival before and after PSM

Baseline characteristics as well as selected variables were enrolled in a univariate analysis between two groups for comparison of both OS and CSS (Table 2). Multivariate analysis found that patients receiving RT harbored both better OS [hazard ratio (HR) 0.840, 95% confidence interval (CI) 0.739–0.954, *P =* 0.007] and CSS (HR 0.847, 95% CI 0.741–0.967, *P =* 0.014) before PSM. Meanwhile, RT was also strongly associated with better survival (OS: HR 0.854, 95% CI 0.736–0.970, *P =* 0.016; CSS: HR 0.848, 95% CI 0.735–0.978, *P =* 0.023) after PSM (Table 3).

**Table 2:** Univariate analyses for prognostic factors before PSM

Variable	OS		OS	
	HR (95% CI)	*P*-Value	HR (95% CI)	*P*-Value
Sex				
Male	1.0		1.0	
Female	0.823 (0.733, 0.924)	<0.001	0.840 (0.744, 0.948)	0.005
Race				
White	1.0		1.0	
Black	1.072 (0.903, 1.273)	0.427	1.077 (0.900, 1.289)	0.419
Other	0.985 (0.735, 1.320)	0.919	0.949 (0.694, 1.298)	0.743
Marital status				
Married	1.0		1.0	
Unmarried	1.065 (0.950, 1.194)	0.281	1.032 (0.915, 1.165)	0.604
Age, years				
<60	1.0		1.0	
≥60 and <80	1.262 (1.105, 1.441)	<0.001	1.214 (1.057, 1.394)	0.006
≥80	1.990 (1.615, 2.451)	<0.001	1.851 (1.484, 2.309)	<0.001
Primary site				
Main bronchus	1.0		1.0	
Upper lobe	0.474 (0.361, 0.621)	<0.001	0.475 (0.358, 0.631)	<0.001
Middle lobe	0.639 (0.453, 0.901)	0.011	0.651 (0.455, 0.933)	0.019
Lower lobe	0.512 (0.385, 0.679)	<0.001	0.514 (0.382, 0.692)	<0.001
Overlapping lesion	0.617 (0.374, 1.018)	0.059	0.616 (0.364, 1.043)	0.071
Grade				
Poorly differentiated	1.0		1.0	
Undifferentiated	1.120 (0.923, 1.358)	0.250	1.129 (0.920, 1.386)	0.244
Unknown	1.772 (1.564, 2.008)	<0.001	1.833 (1.606, 2.091)	<0.001
Laterality				
Bilateral	1.0		-	
Left	1.829 (0.257, 13.017)	0.547	-	-
Right	1.973 (0.277, 14.034)	0.497	-	-
Tumor size				
<2 cm	1.0		1.0	
≥2 cm and <5 cm	1.603 (1.340, 1.918)	<0.001	1.623 (1.339, 1.968)	<0.001
≥5 cm and <10 cm	2.430 (1.998, 2.955)	<0.001	2.563 (2.082, 3.155)	<0.001
≥10 cm	3.465 (2.604, 4.610)	<0.001	3.874 (2.888, 5.197)	<0.001
Unknown	3.882 (3.091, 4.875)	<0.001	4.236 (3.337, 5.377)	<0.001
AJCC stage				
IA	1.0		1.0	
IB	1.138 (0.861, 1.502)	0.364	1.296 (0.944, 1.778)	0.109
IIA	1.569 (0.925, 2.661)	0.094	1.994 (1.143, 3.478)	0.015
IIB	2.345 (1.687, 3.261)	<0.001	2.786 (1.933, 4.014)	<0.001
IIIA	2.309 (1.763, 3.024)	<0.001	2.909 (2.154, 3.929)	<0.001
IIIB	3.769 (2.918, 4.869)	<0.001	4.460 (3.339, 5.957)	<0.001
IV	6.918 (5.566, 8.597)	<0.001	8.778 (6.839, 11.266)	<0.001
Surgery				
No surgery	1.0		1.0	
Segmentectomy/wedge resection	0.328 (0.262, 0.411)	<0.001	0.298 (0.233, 0.380)	<0.001
Lobectomy/bilobectomy	0.200 (0.171, 0.234)	<0.001	0.176 (0.148, 0.209)	<0.001
Pneumonectomy	0.316 (0.229, 0.434)	<0.001	0.290 (0.205, 0.410)	<0.001
Chemotherapy				
No/unknown	1.0		1.0	
Yes	1.010 (0.900, 1.133)	0.870	1.074 (0.951, 1.213)	0.247

**Table 3:** Multivariate analyses with or without RT in patients before and after PSM; model adjusted for age, gender, race, marital status, primary site, grade, laterality, tumor size, AJCC stage, surgery and chemotherapy.

Variable	Before PSM		After PSM	
	HR (95% CI)	*P*-Value	HR (95% CI)	*P*-Value
OS				
No RT	1.0		1.0	
RT	0.840 (0.739, 0.954)	0.007	0.854 (0.736, 0.970)	0.016
CSS				
No RT	1.0		1.0	
RT	0.847 (0.741, 0.967)	0.014	0.848 (0.735, 0.978)	0.023

**Fig. 3.**
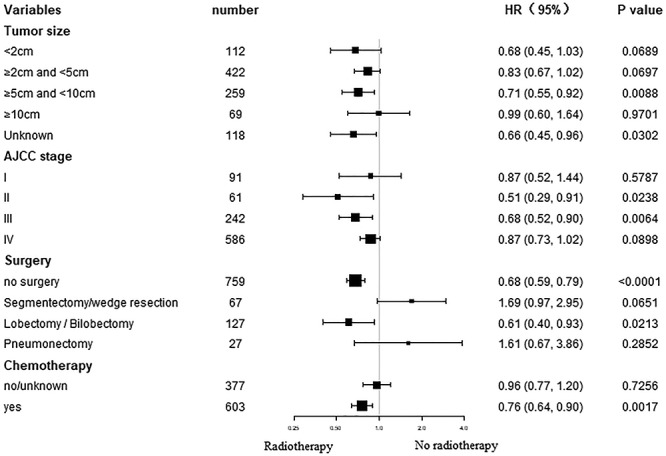
Forest plot of subgroup analysis for OS after 1:1 PSM.

### Subgroup analysis for OS and CSS after PSM

**Fig. 4.**
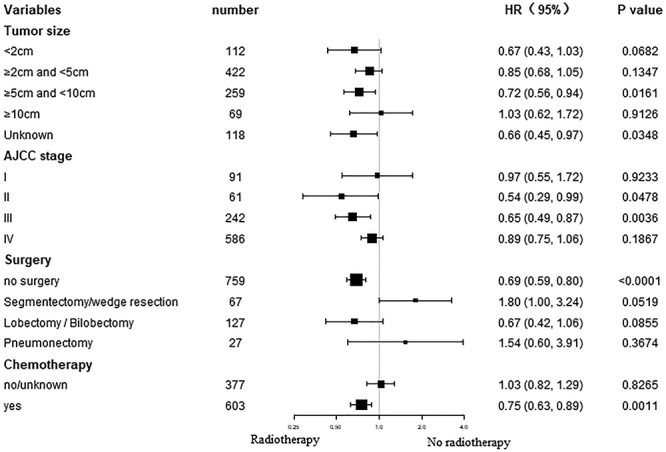
Forest plot of subgroup analysis for CSS after 1:1 PSM.

Subgroup analysis of OS and CSS demonstrated that most study populations could benefit from RT with regard to survival (Figs 3 and 4). AJCC stage II and III, tumor size 5–10 cm, no surgery or received chemotherapy patients could significantly benefit from RT in terms of OS and CSS (all *P* < 0.05).

## DISCUSSION

To our knowledge, this is the first large-population-based study to investigate the role of RT in treating pulmonary LCNEC by using PSM. Consequently, after performing both multivariate regression and PSM analyses, we demonstrated that RT could provide significant survival benefit for patients with pulmonary LCNEC, especially for those with stage II and III.

Pulmonary LCNEC is biologically aggressive with poor prognosis [12]. The 5-year OS in patients receiving resection of LCNEC has been demonstrated to vary from 13 to 57% [4, 13, 14]. Our study found that the 5-year CSS and OS rates of pulmonary LCNEC were 21.2 and 17.3%, respectively. This was a lower survival rate compared to those in previous studies, possibly due to the fact that most of the population included were in stage IV (*n* = 681, 46.0%).

The role of RT in localized or advanced pulmonary LCNEC treatment remains undefined [15–17]. Dresler *et al*. [18] demonstrated that patients with resected LCNEC failed to gain survival benefit from postoperative chemotherapy, RT or both. In contrast, Shimada *et al*. [19] reported comparable outcomes of overall response rate to initial chemotherapy or chemo-RT as well as survival outcomes between high-grade neuroendocrine carcinoma (probable LCNEC) and SCLC. In this study, we found that RT is a significantly protective factor for pulmonary LCNEC. In addition, RT for patients with stage II and III pulmonary LCNEC can significantly improve the survival rate. Moreover, patients with tumor size 5–10 cm, no surgery, or who received chemotherapy could also benefit significantly from RT.

There are certain limitations to this study. Firstly, as in all observational research, it is almost impossible to avoid bias. Although the PSM method could attenuate the bias resulting from unevenly distributed measured covariates, the bias originating from unmeasured ones is unavoidable. Secondly, although 12 variables were involved, there are still some variables that SEER does not include, such as the chemotherapy regimen, surgical margin status and vascular invasion. Thirdly, the definition of RT in the SEER dataset is RT administration during the first course of cancer-directed therapy, without data concerning dose or intended target. Therefore, we had no information about the radiation dose timing, intent, methods, side effects and second-line chemotherapy, which may all have contributed to study bias. Instead of showing it in detail, we aimed to illustrate the general survival advantage of RT in pulmonary LCNEC patients. Thus, the currently accessible information in the SEER dataset could readily satisfy our study requirements. In other words, we had no intention of examining the specific types, dose, timing, intent or methods of RT in pulmonary LCNEC. Nevertheless, the study population was extracted from a national dataset, which could decrease the potential selection bias to some extent. As both multivariable and PSM analyses were performed, while both CSS and OS results did not alter significantly, the findings should be valid and stable.

## CONCLUSION

Our findings based on the SEER database support that RT could provide survival benefit in patients with pulmonary LCNEC, especially for those with stage II and III, tumor size 5-10 cm, with no surgery or who received chemotherapy.

## References

[1] Fasano M, Della Corte CM, Papaccio F et al. Pulmonary large-cell neuroendocrine carcinoma: From epidemiology to therapy. J Thorac Oncol 2015;10:1133–41. 26039012 10.1097/JTO.0000000000000589 PMC4503246

[2] Travis WD, Linnoila RI, Tsokos MG et al. Neuroendocrine tumors of the lung with proposed criteria for large-cell neuroendocrine carcinoma. An ultrastructural, immunohistochemical, and flow cytometric study of 35 cases. Am J Surg Pathol 1991;15:529–53. 1709558 10.1097/00000478-199106000-00003

[3] Rekhtman N Neuroendocrine tumors of the lung: An update. Arch Pathol Lab Med 2010;134:1628–38. 21043816 10.5858/2009-0583-RAR.1

[4] Varlotto JM, Medford-Davis LN, Recht A et al. Should large cell neuroendocrine lung carcinoma be classified and treated as a small cell lung cancer or with other large cell carcinomas? J Thorac Oncol 2011;6:1050–8. 21566535 10.1097/JTO.0b013e318217b6f8

[5] Iyoda A, Azuma Y, Sano A Neuroendocrine tumors of the lung: Clinicopathological and molecular features. Surg Today 2020. 10.1007/s00595-020-01988-7 32193632

[6] Mazieres J, Daste G, Molinier L et al. Large cell neuroendocrine carcinoma of the lung: Pathological study and clinical outcome of 18 resected cases. Lung Cancer (Amsterdam, Netherlands) 2002;37:287–92. 10.1016/s0169-5002(02)00099-5 12234698

[7] Zombori T, Juhasz-Nagy G, Tiszlavicz L et al. Large-cell neuroendocrine carcinoma of the lung - challenges of diagnosis and treatment. Orv Hetil 2020;161:313–9. 32073294 10.1556/650.2020.31581

[8] Duggan MA, Anderson WF, Altekruse S et al. The surveillance, epidemiology, and end results (SEER) program and pathology: Toward strengthening the critical relationship. Am J Surg Pathol 2016;40:e94–e102. 27740970 10.1097/PAS.0000000000000749 PMC5106320

[9] Cronin KA, Ries LA, Edwards BK The surveillance, epidemiology, and end results (SEER) program of the National Cancer Institute. Cancer 2014;120:3755–7. 25412387 10.1002/cncr.29049

[10] Little RJ, Rubin DB Causal effects in clinical and epidemiological studies via potential outcomes: Concepts and analytical approaches. Annu Rev Public Health 2000;21:121–45. 10884949 10.1146/annurev.publhealth.21.1.121

[11] Pattanayak CW, Rubin DB, Zell ER Propensity score methods for creating covariate balance in observational studies. Rev Esp Cardiol 2011;64:897–903. 21872981 10.1016/j.recesp.2011.06.008

[12] Iyoda A, Hiroshima K, Moriya Y et al. Postoperative recurrence and the role of adjuvant chemotherapy in patients with pulmonary large-cell neuroendocrine carcinoma. J Thorac Cardiovasc Surg 2009;138:446–53. 19619794 10.1016/j.jtcvs.2008.12.037

[13] Younossian AB, Brundler MA, Totsch M Feasibility of the new WHO classification of pulmonary neuroendocrine tumours. Swiss Med Wkly 2002;132:535–40. 12508138 10.4414/smw.2002.09880

[14] Liang R, Chen TX, Wang ZQ et al. A retrospective analysis of the clinicopathological characteristics of large cell carcinoma of the lung. Exp Ther Med 2015;9:197–202. 25452802 10.3892/etm.2014.2075 PMC4247287

[15] Hiroshima K, Mino-Kenudson M Update on large cell neuroendocrine carcinoma. Translational Lung Cancer Research 2017;6:530–9. 29114469 10.21037/tlcr.2017.06.12 PMC5653527

[16] Jiang Y, Lei C, Zhang X et al. Double-edged role of radiotherapy in patients with pulmonary large-cell neuroendocrine carcinoma. J Cancer 2019;10:6422–30. 31772675 10.7150/jca.32446 PMC6856741

[17] Lo H, Abel S, Finley G et al. Surgical resection versus stereotactic body radiation therapy in early stage bronchopulmonary large cell neuroendocrine carcinoma. Thorac Cancer 2020;11:305–10. 31860940 10.1111/1759-7714.13260 PMC6997021

[18] Dresler CM, Ritter JH, Patterson GA et al. Clinical-pathologic analysis of 40 patients with large cell neuroendocrine carcinoma of the lung. Ann Thorac Surg 1997;63:180–5. 8993262 10.1016/s0003-4975(96)01058-2

[19] Shimada Y, Niho S, Ishii G et al. Clinical features of unresectable high-grade lung neuroendocrine carcinoma diagnosed using biopsy specimens. Lung Cancer (Amsterdam, Netherlands) 2012;75:368–73. 10.1016/j.lungcan.2011.08.012 21920624

